# Differential responses of scirrhous and well-differentiated gastric cancer cells to orthotopic fibroblasts.

**DOI:** 10.1038/bjc.1996.496

**Published:** 1996-10

**Authors:** M. Yashiro, Y. S. Chung, T. Kubo, F. Hato, M. Sowa

**Affiliations:** First Department of Surgery, Osaka City University Medical School, Japan.

## Abstract

Scirrhous gastric cancer cells proliferate rapidly with fibrosis, when the cancer cells invade into the submucosa of the stomach. To investigate the mechanisms responsible for the rapid proliferation, the growth interaction between gastric cancer cells and fibroblasts was examined. Human gastric cancer cell lines established from scirrhous carcinoma or well-differentiated adenocarcinoma were used. Human fibroblast cell lines were obtained from various organs. The growth interaction between gastric cancer cells and fibroblasts was examined by calculating the number of cancer cells or by measuring [3H]thymidine incorporation of cancer cells. Gastric fibroblasts specifically stimulated the growth of scirrhous gastric cancer cells, but not that of well-differentiated adenocarcinoma cells. The growth factor(s) produced from gastric fibroblasts were then partially purified and characterised. The growth-promoting factor(s) had apparent molecular weights of 10000 dalton and was sensitive both to heat and proteinase treatment. No inhibition for the factor(s) was achieved with defined anti-growth factor antibodies. In this study, differential responses of scirrhous and well-differentiated gastric cancer cells to orthotopic fibroblasts were shown. Rapid proliferation of scirrhous gastric carcinoma should be partly controlled by orthotopic fibroblasts. The growth factor(s) from gastric fibroblasts, which was distinct from various defined growth factors such as epidermal growth factor (EGF), basic fibroblast growth factor (b-FGF), transforming growth factor-alpha (TGF-alpha), keratinocyte growth factor (KGF), vascular endothelial growth factor (VEGF), insulin-like growth factor I (IGF-I), hepatocyte growth factor (HGF), platelet-derived growth factor (PDGF) and transforming growth factor beta 1 (TGF-beta 1) may play an important role in the progression of scirrhous gastric cancer cells.


British Journal of Cancer (1996) 74, 1096-1103
© 1996 Stockton Press All rights reserved 0007-0920/96 $12.00

### Differential responses of scirrhous and well-differentiated gastric cancer cells to orthotopic fibroblasts

M Yashiro[1], Y-S Chung[1], T Kubo[1], F Hato[2] and M Sowa[1]

[1]*First Department of Surgery and* [2]*Second Deparment of Physiology, Osaka City University Medical School, 1-5-7 Asahimachi, Abeno-Ku, Osaka 545, Japan.*

**Summary** Scirrhous gastric cancer cells proliferate rapidly with fibrosis, when the cancer cells invade into the submucosa of the stomach. To investigate the mechanisms responsible for the rapid proliferation, the growth interaction between gastric cancer cells and fibroblasts was examined. Human gastric cancer cell lines established from scirrhous carcinoma or well-differentiated adenocarcinoma were used. Human fibroblast cell lines were obtained from various organs. The growth interaction between gastric cancer cells and fibroblasts was examined by calculating the number of cancer cells or by measuring [$^3$H]thymidine incorporation of cancer cells. Gastric fibroblasts specifically stimulated the growth of scirrhous gastric cancer cells, but not that of well-differentiated adenocarcinoma cells. The growth factor(s) produced from gastric fibroblasts were then partially purified and characterised. The growth-promoting factor(s) had apparent molecular weights of 10 000 dalton and was sensitive both to heat and proteinase treatment. No inhibition for the factor(s) was achieved with defined anti-growth factor antibodies. In this study, differential responses of scirrhous and well-differentiated gastric cancer cells to orthotopic fibroblasts were shown. Rapid proliferation of scirrhous gastric carcinoma should be partly controlled by orthotopic fibroblasts. The growth factor(s) from gastric fibroblasts, which was distinct from various defined growth factors such as epidermal growth factor (EGF), basic fibroblast growth factor (b-FGF), transforming growth factor-$\alpha$ (TGF-$\alpha$), keratinocyte growth factor (KGF), vascular endothelial growth factor (VEGF), insulin-like growth factor I (IGF-I), hepatocyte growth factor (HGF), platelet-derived growth factor (PDGF) and transforming growth factor $\beta$1 (TGF-$\beta$1) may play an important role in the progression of scirrhous gastric cancer cells.

**Keywords:** scirrhous gastric cancer; well-differentiated gastric cancer; fibroblast; growth interaction; growth factor

Human scirrhous gastric carcinoma (diffusely infiltrating carcinoma or Borrman's type IV carcinoma) is characterised by cancer cell infiltration and proliferation with extensive fibrosis in the stroma (Tahara, 1990). Although the prognosis of gastric cancer has recently improved, that of scirrhous gastric cancer has not (Kiyasu *et al.*, 1981). One of the reasons for the poor prognosis of this type of cancer is the difficulty of diagnosing it at an early stage, in part because of the rapid proliferation of the cancer cells. When scirrhous gastric cancer cells invade into submucosa of stomach, the cancer cells proliferate rapidly with fibrosis. The mechanisms responsible for the rapid proliferation are not understood clearly. The typical histological findings of scirrhous gastric carcinoma suggest that its development may be controlled by intercellular interactions between the cancer cells and the stroma cells such as fibroblasts. Recently, several studies have been published on the effect of fibroblasts on the production of the extracellular matrix of gastric cancer cells (Naito *et al.*, 1984; Yamamoto *et al.*, 1990). However, there has been no published report about the growth effect of gastric fibroblasts on scirrhous gastric cancer cells. Therefore, we examined the growth interaction between gastric cancer cells and fibroblasts derived from different organs, and partly purified and characterised a growth factor(s) for scirrhous gastric carcinoma.

### Materials and methods

### *Cell types and cell culture*

The human gastric cancer cell lines, OCUM-2M (poorly differentiated adenocarcinoma) (Yashiro *et al.*, 1995), OCUM-1 (poorly differentiated adenocarcinoma), KATO-III (signet-ring cell carcinoma) (Sekiguchi *et al.*, 1978), MKN-28 (well-differentiated adenocarcinoma) (Hojo, 1977), MKN-74 (well-differentiated adenocarcinoma) (Hojo, 1977), were cultivated in medium (see below) in a 100-mm culture dish (Falcon, Lincoln Park, NJ, USA), and incubated at 37°C in a humidified atmosphere of 5% carbon dioxide in air. OCUM-2M, OCUM-1 and KATO-III were derived from scirrhous gastric carcinoma.

Human fibroblast cell lines were obtained from various organs. Original organ of each fibroblast cell line is presented in Table I. A fibroblast cell line, NF-8, and a scirrhous gastric cancer cell line, OCUM-2M, were obtained from the same patient (Yashiro *et al.*, 1994). NF-1, NF-Eso, NF-Je and NF-Co were obtained from the same patient. The other fibroblast cell lines were derived from different patients. HS-27F was obtained from the American Type Culture Collection (Rockville, MD, USA), and the other fibroblast cell lines were derived from normal tissues of each organ in our laboratory, as follows. Briefly, each tissue specimen was excised under aseptic conditions and minced with forceps and scissors. Pieces of each tissue were cultivated in medium in a 100-mm culture dish (Falcon) and incubated in humidified incubators at 37°C in an atmosphere of 5% carbon dioxide and 95% air. The fibroblasts gradually grew in a monolayer. When confluent, the fibroblasts were collected and transferred to another culture dish every 5–7 days. The fibroblast origin was verified by immunostaining with two monoclonal antibodies against vimentin and human fibroblast (Dako, Glostrup, Denmark).

The culture medium was composed of Dulbecco's modified Eagle medium (DMEM) (Bioproducts, Walkersville, MD, USA) with 2% heat-inactivated fetal calf serum (FCS) (Gibco, Grand Island, NY, USA), 100 IU $ml^{-1}$ penicillin (ICN Biomedicals, Costa Mesa, CA, USA), 100 $\mu g\ ml^{-1}$ streptomycin (ICN Biochemicals), 2 mM glutamine (Bioproducts) and 0.5 mM sodium pyruvate (Bioproducts).

Correspondence: M Yashiro
Received 16 October 1995; Revised 17 April 1996; accepted 24 April 1996

**Table I** Original organ of fibroblast cell line

| *Fibroblast cell line* | *Original organ* |
|---|---|
| NF-8 | Stomach |
| NF-1 | Stomach |
| NF-Eso | Oesophagus |
| NF-Je | Jejum |
| NF-Co | Colon |
| NF-Liver | Liver |
| NF-Pa | Parotis |
| NF-Ma | Mamma |
| NF-Ov | Ovary |
| HS-27F | Foreskin |

*Preparation of serum-free conditioned media*

Serum-free conditioned medium (SF-CM) from fibroblasts was prepared as follows. $5.0 \times 10^5$ fibroblasts were seeded into 100-mm plastic dishes with 10 ml of DMEM containing 2% FCS, and incubated at 37°C for 3 days. To obtain the SF-CM, the fibroblasts were washed twice with Dulbecco's phosphate-buffered saline (PBS) and then incubated for 2 days with 1 ml of DMEM. The number of fibroblasts in each dish was approximately $2 \times 10^6$ cells at the collection of SF-CM. The SF-CM was collected and centrifuged at 1000 *g* for 5 min, passed through filters (pore size 0.45 $\mu$m; Kurabo, Osaka, Japan) and stored at $-20$°C until use. The fibroblasts were used before the 15th passage in culture. Proliferative ability measured as doubling time was not different among the fibroblast cell lines.

*Effect of fibroblasts on the growth of gastric cancer cells*

The proliferation of the gastric cancer cells was determined by calculating the number of cancer cells or by measuring [$^3$H]thymidine incorporation.

The number of cancer cells was calculated following the addition of SF-CM from fibroblasts using a Coulter counter (Industrial D; Coulter Electronics, Luton, UK). To determine the optimal concentration of SF-CM for its growth-promoting activity, the kinetics and serum dependency of the activity produced from NF-8 cells were examined by culturing OCUM-2M cells. OCUM-2M cells were cultured in 24-well plates for 3 days in the presence of varying concentrations of SF-CM from NF-8 cells. Since the activity for OCUM-2M was evident following the addition of 25% SF-CM with 1–2% FCS (see Figure 1), the growth-promoting assay was conducted at 25% SF-CM. Briefly, 250 $\mu$l of SF-CM was added to 750 $\mu$l of tumour cell suspension ($1 \times 10^4$ cells per well) with 2% FCS in each well of 24-well dishes, and incubated. The number of cells was counted at various time points using a Coulter counter. Serum-free medium instead of active fraction was used as a control.

The effect of fibroblasts on DNA synthesis of gastric cancer cells was determined by measuring [$^3$H]thymidine incorporation. Briefly, 750 $\mu$l of the tumour cell suspension ($1 \times 10^5$ cells per well) with 2% FCS was added to 250 $\mu$l of SF-CM in each well of 24-plates, and incubated with a pulse of 1 $\mu$Ci per well of [$^3$H]thymidine (28 Ci mmol$^{-1}$; Amersham, Tokyo, Japan) for 24 at 37°C. As a control, 250 $\mu$l of DMEM was used. The cells were then rinsed and collected on a membrane filter, and the radioactivity incorporated into DNA was determined in a liquid scintillation counter (Aloka, Tokyo, Japan).

*Treatment of serum-free conditioned medium*

The SF-CM obtained from NF-8 was used to characterise the growth-promoting activity. The growth activity was studied by culturing OCUM-2M cells as target. For measurement of heat stability of the growth-promoting activity, SF-CM was heated to 56°C for 30 min, 80°C for 10 min, and 100°C for

**Figure 1** Proliferative effects of conditioned medium from NF-8 cells on OCUM-2M cells. (**a**) OCUM-2M cells were cultured for 3 days in the presence of varying doses of the serum-free conditioned medium with 2% FCS. OCUM-2M cells were significantly increased following the addition of 10–50% SF-CM. (**b**) The growth of OCUM-2M cells with 25% SF-CM (●) was significantly increased in the presence of 0.3–20% FCS compared with control (■). The growth activity was evident with 1–2% FCS. The results are presented as the mean of three samples and the bars indicated the s.d. **P*<0.05; ***P*<0.01 *vs* control.

30 min. The susceptibility of the activity to proteases was examined by incubation of the SF-CM with 1 unit ml$^{-1}$ of proteases, trypsin (Sigma, St Louis, MO, USA), $\alpha$-chymotrypsin (Sigma), or proteinase K (Sigma) at 37°C for 24 h. All samples were passed through filters (Kurabo). To determine whether the growth-promoting factor possessed heparin affinity, we examined the activity of SF-CM loaded onto the heparin affinity column ECHONO-Pac heparin cartridge (Bio-Rad, Richmond, CA, USA). Treated SF-CM (250 $\mu$l) was added to 750 $\mu$l of OCUM-2M cell suspension ($1 \times 10^4$ cells per well) with 2% FCS in each well of 24-well dishes, and cultured for 3 days. The growth-promoting activity of the treated SF-CM was determined by calculating the number of OCUM-2M cells. The growth-promoting activity (% control) was calculated as:

$$\text{Growth} - \text{promoting activity (\% control)}$$

$$= \frac{\text{number of cells cultured in medium with samples}}{\text{number of cells cultured in medium alone}} \times 100 - 100.$$

**Figure 2** Effect of SF-CM from fibroblasts on the growth of various cancer cells. SF-CM from gastric fibroblasts (NF-1, NF-8) significantly increased the number of scirrhous gastric cancer cells (OCUM-2M, OCUM-1, KATO-III) but not that of well-differentiated adenocarcinoma cells (MKN-28, MKN-74). SF-CM from foreskin fibroblasts (HS-27F) did not stimulate the number of any cancer cells. The results are presented as the mean of three samples and the bars indicate the s.d. $^{*}P<0.01$ *vs* control.

*Ion-exchange chromatography*

The SF-CM from NF-8 was applied to a TSK-gel DEAE-5PW column (75 × 7.5 mm; Tosoh, Tokyo, Japan) equilibrated with PBS. The column was washed with 20 ml PBS and the bound protein was eluted with a linear gradient of 0–0.8 M sodium chloride in the PBS. Eluted protein was detected by UV absorption at 280 nm. All manipulations were carried out at room temperature. Aliquots of 250 $\mu$l of each fraction were added to 750 $\mu$l of OCUM-2M cell suspension ($1 \times 10^4$ cells per well) with 2% FCS in each well of 24-well dishes, and growth-promoting activity was determined by calculating the number of cancer cells. Active fractions (20 ml) were then combined and concentrated to about 2 ml by ultrafiltration.

*Gel filtration chromatography*

The concentrated active fractions were applied to a TSK-gel G2000SWXL column (300 × 7.5 mm; Tosoh) equilibrated with PBS. The fractions were collected at a flow rate of 1 ml $min^{-1}$. The wavelength of the detector was set to 280 nm. All manipulations were carried out at room temperature. Ferritin ($M_r$ 450 000), bovine serum albumin ($M_r$ 67 000), ovalbumin ($M_r$ 45 000), chymotrypsinogen A ($M_r$ 25 000), cytochrom C ($M_r$ 12 300) and insulin chain B ($M_r$ 3500) were used as the standard samples for the molecular weight calibration. The standard samples were purchased from Serva Feinbiochemica, Heidelberg, Germany. An aliquot of 750 $\mu$l of OCUM-2M cell suspension ($1 \times 10^4$ cells per well) with 2% FCS was inoculated into each well of 24-well plates (Falcon) with a pulse of 250 $\mu$l of each fraction, and incubated. After 3 days the number of cancer cells was counted.

*Effect of defined growth factors on the growth of OCUM-2M cells*

We examined the effect of various defined growth factors, including EGF (Gibco), b-FGF (Austral Biologics, San Ramon, CA, USA) KGF (UBI, Lake Placid, NY, USA), VEGF (Pepro Tec, Rocky Hill, NJ, USA), TGF-$\alpha$ (Becton Dickinson Labware, Mountain View, CA, USA), IGF-I (Mallinckrodt, St Louis, MO, USA), PDGF-AA (Austral Biologicals), HGF (Becton Dickinson Labware), and TGF-$\beta$1 (King Brewing, Kakogawa, Japan) which are thought to affect the growth of gastric carcinoma (Yoshida *et al.*, 1989, 1990; Yasui *et al.*, 1988; Tanimoto *et al.*, 1991; Hattori *et al.*, 1994; Shibamoto *et al.*, 1992; Ito *et al.*, 1992) on the growth of OCUM-2M cells. An aliquot of 1 ml of OCUM-2M cell suspension ($1.0 \times 10^4$ cells $ml^{-1}$) was inoculated into each well of 24-well plates (Falcon) with various concentrations of the defined growth factors, and incubated. The number of OCUM-2M cells was counted at various time points.

*Effect of anti-growth factor antibodies on the growth activity of conditioned medium from fibroblasts*

We used neutralising antibodies for several growth factors including anti-human EGF antibody (Oncogene Science, Uniondale, NY, USA), anti-human b-FGF antibody (Wako, Tokyo, Japan) (Hori *et al.*, 1991), anti-TGF-$\alpha$ antibody (Pepro Tec), anti-HGF antibody (Sigma), rabbit IgG standard (Zymed, San Francisco, CA, USA) and mouse IgG standard (Tago, Burlingame, CA, USA). These antibodies were reconstituted in 0.1 g BSA per 100 ml of serum-free medium. Effect of the neutralising antibodies against their respective ligands was examined in experimental study (see Table III). Antibody solutions of 4, 20, 100 and 400 $\mu g\ ml^{-1}$ were prepared and mixed 1:1 (v/v) with the peak growth activity fraction 22 by high-performance liquid chromatography (HPLC) (see Figure 3b). A 500 $\mu l$ portion of OCUM-2M cell suspension ($1 \times 10^4$ cells per well) with 2% FCS was inoculated into each well of 24-well plates (Falcon). Antibody solutions (500 $\mu l$) were added to each well and incubated. Final solutions contained 25% fraction sample and antibody concentrations of 1, 5, 25 or 100 $\mu g\ ml^{-1}$. After 3 days the number of OCUM-2M cells was counted. Serum-free medium instead of active fraction was used as a control.

*Statistical analysis*

Data were analysed statistically using Student's *t*-test. A *P*-value less than 0.05 was considered statistically significant.

### Results

*Growth-promoting activity of serum-free conditioned medium from fibroblasts for various gastric cancer cells*

The activity for OCUM-2M cells was evident following the addition of 25% SF-CM with 1–2% FCS (Figure 1); the

**Figure 3** Effect of SF-CM from fibroblasts on DNA synthesis of gastric cancer cells. (**a**) The [$^3$H]thymidine incorporation of OCUM-1 and KATO-III cells was significantly enhanced 130–180% by SF-CM from orthotopic fibroblasts (NF-1, NF-8), while that of MKN-28 and MKN-74 cells was not enhanced by SF-CM from any fibroblasts. (**b**) The [$^3$H]thymidine incorporation of OCUM-2M cells was significantly enhanced 130–150% by SF-CM from orthotopic fibroblasts (NF-1, NF-8) compared with control, while that of OCUM-2M cells was not enhanced by SF-CM from various ectopic fibroblasts. The results are presented as the mean of three independent experiments and the bars indicate the s.d. *$P<0.05$; **$P<0.01$ *vs* control.

growth-promoting assay was then conducted at this concentration. The three scirrhous gastric cancer cell lines grew floating but not anchorage dependent in the culture medium and were not adherent to dishes following the addition of the SF-CM. The SF-CM from gastric fibroblasts (NF-1, NF-8) significantly increased the number of scirrhous gastric cancer cells (OCUM-2M, OCUM-1, KATO-III) after 60 h in culture but not that of well-differentiated adenocarcinoma cells (MKN-28, MKN-74). The SF-CM from foreskin fibroblasts (HS-27F) did not increase the number of any cancer cells (Figure 2). The SF-CM from orthotopic fibroblasts (NF-1, NF-8) specifically stimulated the DNA synthesis of scirrhous gastric cancer cells by 130–180% compared with control, but not well-differentiated adenocarcinoma cells. The SF-CM from various ectopic fibroblasts did not stimulate the DNA synthesis of any type of gastric cancer cells (Figure 3).

**Table II** Biochemical characterisations of growth-promoting activity in the serum-free conditioned medium from NF-8 cells[a]

| | *Growth-promoting activity (% control)* | *Inhibition (%)* |
|---|---|---|
| Heat treatment | | |
| Untreated | 32 | |
| 56°C for 30 min | 11 | 66 |
| 80°C for 10 min | 2 | 93.7 |
| 100°C for 30 min | 0 | 100 |
| Enzyme treatment | | |
| Untreated | 52 | |
| Trypsin (1 unit $ml^{-1}$) | 0 | 100 |
| α-Chymotrypsin (1 unit $ml^{-1}$) | 0 | 100 |
| Proteinase K (1 unit $ml^{-1}$) | 0 | 100 |
| Heparin affinity | | |
| Before heparin chromatography | 48 | |
| After heparin chromatography | 45 | 6 |

[a]Serum-free conditioned medium from NF-8 was subjected to different treatments as described in Materials and methods. The

**Figure 4** Purification of the growth-promoting activity. (**a**) Cation-exchange chromatography. The SF-CM from NF-8 was applied to a TSK-gel DEAE-5PW column. Elution of protein was monitored by absorption at 280 nm. The growth-promoting activity of each fraction was examined by calculating the number of OCUM-2M cells. Peak activity was eluted at 160 mM sodium chloride, fractions 9 and 10. (**b**) Gel filtration chromatography of the SF-CM from NF-8. The SF-CM was applied to a TSK-gel G2000SWXL column and eluted with PBS. The growth-promoting activity was determined by calculating the number of OCUM-2M cells. Calculated molecular weight of the major peak was 10 000 dalton. Arrowheads indicate positions of standard molecular markers. Molecular weight markers included: ferritin ($M_r$ 450 000), bovine serum albumin ($M_r$ 67 000), ovalbumin ($M_r$ 45 000), chymotrypsinogen A ($M_r$ 25 000), cytochrom C ($M_r$ 12 300) and insulin chain B ($M_r$ 3500).

**Figure 5** Effect of defined growth factors on the growth of OCUM-2M cells. (**a**) OCUM-2M cells were cultured with various defined growth factors in concentrations ranging from 0.1–100 ng $ml^{-1}$, and then cell proliferation was determined by calculating the number of cancer cells after 72 h in culture. EGF, VEGF, TGF-α, KGF and b-FGF significantly stimulated OCUM-2M cell growth in concentrations ranging from 10–100 ng $ml^{-1}$ compared with control cells. IGF-I, PDGF and HGF had no significant effect on the growth of OCUM-2M cells. TGF-*β* decreased the growth of OCUM-2M cells. (**b**) OCUM-2M cells were cultured with 10 ng $ml^{-1}$ growth factors, and cell proliferation was determined at various time points. The growth of OCUM-2M cells was stimulated by EGF, VEGF, TGF-α, b-FGF and KGF after 48 h culture. The growth effect was evident after 72 h culture. Points, means of three samples and the bars indicate the s.d.

*Characterisation of the growth-promoting activity*

The effects of various treatments on the growth-promoting activity of the conditioned medium are shown in Table II. Protein concentrations in SF-CM from fibroblasts were measured by a Bio-Rad protein assay kit (Bio-Rad, Richmond, VA, USA) using BSA as a standard. Protein concentration in each SF-CM was 40.6–49.3 $\mu g\ ml^{-1}$ per $2\times10^6$ cells. The activity was partially lost when heated at 56°C for 30 min and completely lost when heated at 80°C for 10 min. Treatment with trypsin, α-chymotrypsin, or proteinase K also destroyed the activity completely. The growth-promoting activity of SF-CM was retained even after heparin chromatography (Table II).

*Purification of the growth-promoting activity*

The SF-CM from NF-8 was applied to a TSK-gel DEAE-5PW column. Peak activity was eluted at 160 mM sodium chloride (Figure 4a). The active fractions 9 and 10 were concentrated and applied to a TSK-gel G2000SWXL column. A peak of growth-promoting activity was observed in fraction 22. From calculation of molecular weight of the polypeptide using the standard samples, it was estimated that the apparent molecular weight of the major peak was 10 000 dalton (Figure 4b). The growth of MKN-28 cells was not stimulated following the addition of fraction 22 (data not shown).

*Effect of defined growth factors on the growth of OCUM-2M cells*

To identify possible mitogens involved in OCUM-2M cell growth, we investigated the dose–response relationship between OCUM-2M cells and defined growth factors, including EGF, VEGF, TGF-α, IGF-I, KGF, b-FGF, PDGF-AA, HGF and TGF-β. OCUM-2M cells were cultured with these growth factors in concentrations ranging from 0.1–100 ng $ml^{-1}$. EGF, TGF-α, VEGF, KGF and b-FGF significantly stimulated OCUM-2M cell growth in concentrations ranging from 10 to 100 ng $ml^{-1}$ after 72 h culture. IGF-I, PDGF and HGF had no significant effect on the growth of OCUM-2M cells. TGF-β decreased the growth of OCUM-2M cells (Figure 5a). The growth effect was evident after 72 h culture (Figure 5b).

*Effect of anti-growth factor antibodies on the growth activity of conditioned medium from gastric fibroblasts*

To determine the relation between the growth activity of SF-CM and the defined growth factors which stimulated OCUM-2M cell growth, we tested whether neutralising antibodies against EGF, b-FGF and PDGF-AA were able to neutralise the growth-stimulating activity of the HPLC fraction 22. Bioactivity of each antibody in OCUM-2M cells was demonstrated in Table III. The growth activity of the fraction was not inhibited by any neutralising antibody (Table IV).

### Discussion

In scirrhous gastric carcinoma, which is characterised by extensive carcinoma cell infiltration and proliferation with fibrosis, it is plausible that fibroblasts could affect the progression of the cancer cells from the standpoint of its characteristic histological findings. However, the growth interaction between gastric cancer cells and gastric fibroblasts has not been reported. In the present study, we have reported the organ-specific growth interaction between gastric cancer cells and fibroblasts. Besides NF-1 and NF-8, another five stomach-derived fibroblast cell lines also significantly stimulated the growth of scirrhous gastric cancer cells but not well-differentiated adenocarcinoma cells (data not shown). It was considered that gastric fibroblasts might specifically stimulate the growth of scirrhous gastric cancer cells in a

**Table III** Bioactivity[a] of neutralising antibodies against 10 ng $ml^{-1}$ of EGF, TGF-α, b-FGF and VEGF

| *Antibody* | *Growth-promoting activity (% control)* | *Bioactivity (% inhibition)* |
|---|---|---|
| Anti-EGF antibody | | |
| Control | 29 | |
| 1 µg $ml^{-1}$ | 26 | 4 |
| 5 µg $ml^{-1}$ | 26 | 4 |
| 25 µg $ml^{-1}$ | 9 | 69 |
| Anti-TGF-α antibody | | |
| Control | 25 | |
| 10 µg $ml^{-1}$ | 25 | 0 |
| 25 µg $ml^{-1}$ | 24 | 4 |
| 100 µg $ml^{-1}$ | 3 | 88 |
| Anti-b-FGF antibody | | |
| Control | 18 | |
| 1 µg $ml^{-1}$ | 4 | 78 |
| 5 µg $ml^{-1}$ | 0 | 100 |
| 25 µg $ml^{-1}$ | 0 | 100 |
| Anti-VEGF antibody | | |
| Control | 26 | |
| 10 µg $ml^{-1}$ | 26 | 0 |
| 25 µg $ml^{-1}$ | 24 | 8 |
| 100 µg $ml^{-1}$ | 6 | 77 |

[a]Neutralising antibodies were mixed with OCUM-2M cells which were cultured with each growth factor. The growth activity and bioactivity were determined for OCUM-2M cells as described in Materials and methods.

**Table IV** Effect of neutralising antibodies against EGF, TGF-α, b-FGF and VEGF on the growth activity of fraction 22[a]

| *Antibody* | *Growth-promoting activity (% control)* | *Inhibition (%)* |
|---|---|---|
| Untreated | 78 | 0 |
| Mouse IgG | | |
| 1 µg $ml^{-1}$ | 80 | 0 |
| 10 µg $ml^{-1}$ | 72 | 8 |
| 25 µg $ml^{-1}$ | 70 | 10 |
| Rabbit IgG | | |
| 10 µg $ml^{-1}$ | 75 | 4 |
| 25 µg $ml^{-1}$ | 74 | 5 |
| 100 µg $ml^{-1}$ | 69 | 12 |
| Anti-EGF antibody | | |
| 1 µg $ml^{-1}$ | 76 | 3 |
| 5 µg $ml^{-1}$ | 73 | 7 |
| 25 µg $ml^{-1}$ | 66 | 15 |
| Anti-TGF-α antibody | | |
| 10 µg $ml^{-1}$ | 77 | 1 |
| 25 µg $ml^{-1}$ | 71 | 9 |
| 100 µg $ml^{-1}$ | 71 | 9 |
| Anti-b-FGF antibody | | |
| 1 µg $ml^{-1}$ | 80 | 0 |
| 5 µg $ml^{-1}$ | 70 | 10 |
| 25 µg $ml^{-1}$ | 68 | 13 |
| Anti-VEGF antibody | | |
| 10 µg $ml^{-1}$ | 79 | 0 |
| 25 µg $ml^{-1}$ | 80 | 0 |
| 100 µg $ml^{-1}$ | 80 | 0 |

[a]Neutralising antibodies were mixed with the fractions 22 by HPLC, which stimulated the growth of OCUM-2M cells. The growth activity was determined for OCUM-2M cells as described in Materials and methods.

general principle. The interactions between fibroblasts and cancer cells, including breast cancer cells (Chiquet-Ehrismann et al., 1989), melanoma cells (Enami et al., 1983; Horgan et al., 1987; Cronil et al., 1991), lung cancer cells (Ankrapp and Bevan, 1993), prostate cancer cells (Gleave et al., 1989) and salivary adenocarcinoma cells (Shirasuma et al., 1988), have been reported previously. In this study, however, it was interesting that gastric cancer cells of varying differentiation had differential responses to gastric fibroblasts. Most well-differentiated adenocarcinoma cells proliferate in a medullary pattern, while scirrhous gastric cancer cells proliferate diffusely with extensive fibrosis (Japanese Research Society for Gastric Cancer, 1995). This histological difference in the volume of the stroma might be determined by the response of gastric cancer cells to orthotopic fibroblasts.

The active molecule is considered to be a protein because of its sensitivity to heat treatment and various proteinases. The apparent molecular weight ($M_r$ 10 000) of the growth factor(s) produced from NF-8 cells was estimated by gel filtration HPLC. It has been reported that the progression of gastric carcinoma is associated with several growth factors including EGF, TGF-$\alpha$, b-FGF, VEGF, IGF-I, KGF, HGF, PDGF and TGF-$\beta$ (Yoshida et al., 1990; Yasui et al., 1988; Tanimoto et al., 1991; Hattori et al., 1994; Shibamoto et al., 1992; Ito et al., 1992; Yoshida et al., 1989). Most scirrhous gastric cancer cell lines are known to be stimulated by EGF, b-FGF and KGF (Yoshida et al., 1990; Yasui et al., 1988; Tanimoto et al., 1991; Hattori et al., 1994). The growth of OCUM-2M cells was also stimulated by EGF, TGF-$\alpha$, VEGF, b-FGF and KGF, but not IGF-I, PDGF, HGF and TGF-$\beta$. In general, fibroblasts have been reported to produce growth factors (Ankrapp et al., 1993; Hlatky et al., 1994; Lawrence et al., 1984; Mooradian et al., 1992; Mukai et al., 1989). We examined whether any of the defined growth factors including EGF, VEGF, TGF-$\alpha$, b-FGF and KGF, was the active factor in the conditioned medium from gastric fibroblasts. However, the growth activity of the conditioned medium was not decresed by neutralising antibody against EGF, TGF-$\alpha$, b-FGF and VEGF. In addition, no reactivity with these antibodies in the active fraction was shown by immunoblotting (data not shown). Conditioned medium loaded onto a heparin affinity column had the growth-promoting activity equal to that of the conditioned medium before loading, which indicates that the heparin affinity growth factors such as KGF, b-FGF and VEGF are distinct from the growth activity present in SF-CM. Recently, more than fifty cell growth factors have been evident. In this study, only nine growth factors were examined; however, these factors were almost included in all factors which had been reported previously to be associated with the growth of gastric cancer cells. These findings suggest that the growth factor(s) produced by gastric fibroblasts might be different from the defined factors known to be associated with the progression of gastric carcinoma.

In conclusion, rapid proliferation of scirrhous gastric carcinoma should be partly controlled by gastric fibroblasts. There has been no previous report of a growth factor with growth-promoting activity which depends on the histological type of gastric cancer cells. The growth factior(s) produced by gastric fibroblasts might be a unique factor(s) and may play an important role in the progression of scirrhous gastric cancer.

### Abbreviations

DMEM, Dulbecco's modified Eagle medium; PBS, Dulbecco's phosphate-buffered saline; FCS, fetal calf serum; BSA, bovine serum albumin; EGF, epidermal growth factor; b-FGF, basic fibroblast growth factor; KGF, keratinocyte growth factor; VEGF, vascular endothelial cell growth factor; TGF-$\alpha$, transforming growth factor alpha; IGF-I, insulin growth factor-I; PDGF-AA, platelet-derived growth factor AA homodimer; HGF, hepatocyte growth factor; TGF-$\beta$1, transforming growth factor-$\beta$1; HPLC, high-performance liquid chromatography.

### Acknowledgements

This work was partly supported by grants from the Ministry of Health and Welfare of Japan.

### References

ANKRAPP PD AND BEVAN RD. (1993). Insulin-like growth factor-I and human lung fibroblast-derived insulin-like growth factor-I stimulate the proliferation of human lung carcinoma cells *in vitro*. *Cancer Res.*, **53,** 3399–3404.

CHIQET-EHRISMAN R, KALLA P AND PEARSON AC. (1989). Participation of tenascin and transforming grwoth factor-$\beta$ in reciprocal epithelial–mesenchymal interactions of MCF7 cells and fibroblasts. *Cancer Res.*, **49,** 4322–4325.

CRONIL I, THEODORESCU D, MAN S, HERLYN M, JABROSIC J AND KERBEL RS. (1991). Fibroblast cell interactions with human melanoma cells after tumor cell growth as a function of tumor progression. *Proc. Natl Acad. Sci. USA*, **88,** 6028–6032.

ENAMI J, ENAMI S AND KOGA M. (1983). Growth of normal and neoplastic mouse mammary epithelial cells in primary culture: stimulation by conditioned medium from mouse mammary fibroblasts. *Jpn. J. Cancer Res.*, **74,** 845–853.

GLEAVE M, HSIEH TJ, GAO C, ESCHENBACH CA AND CHUNG LWK. (1989). Acceleration of human prostate cancer growth *in vitro* by factors produced by prostate and bone fibroblasts. *Cancer Res.*, **51,** 3753–3761.

HATTORI Y, ODARGIRI H, NAKATANI H, MIAYZAWA K, NAITO K, SAKAMOTO H, KATOH O, YOSHIDA T, SUGIMURA T AND TERADA M. (1994). K-*sam*, an amplified gene in stomach cancer, is a member of the heparin-binding growth factor receptor genes. *Proc. Natl Acad. Sci. USA*, **87,** 5983–5987.

HLATKY L, TSIONOU C, HAHNFELDT P AND COLEMAN N. (1994). Mammary fibroblasts may influence breast tumor angiogenesis via hypoxia-induced vascular endothelial growth factor up-regulation and protein expression. *Cancer Res.*, **54,** 6083–6086.

HOJO H. (1977). Establishment of cultured cell lines of human stomach cancer origin and their morphological characteristics. *J. Niigata Exp. Med.*, **91,** 737–763.

HORGAN D, JONES LD AND MANSEL ER. (1987). Mitogenicity of human fibroblasts *in vivo* for human breast cancer cells. *Br. J. Surg.*, **74,** 227–229.

HORI A, SASADA R, MATSUNAMI E, NAITO K, SAKURA Y, FUJITA T AND KOZAI Y. (1991). Suppression of solid tumour growth by immunoneutralizing monoclonal antibody against human basic fiboblast growth factor. *Cancer Res.*, **51,** 6180–6184.

ITO M, YASUI W, KYO E, YOKOZAKI H, NAKAYAMA H, ITO H AND TAHARA E. (1992). Growth inhibition of transforming growth factor $\beta$ on human gastric carcinoma cells: receptor and postreceptor signalling. *Cancer Res.*, **52,** 295–300.

JAPANESE RESEARCH SOCIETY FOR GASTRIC CANCER. (1995). *The General Rules for Gastric Cancer Study. Part II. Histological Findings*. 1st English edition. Kanehara: Tokyo.

KIYASU Y, KANESHIMA S AND KOGA S. (1981). Morphogenesis of peritoneal metastasis in human gastric cancer. *Cancer Res.*, **41,** 1236–1239.

LAWRENCE AD, PIRCHER R, KRYCEVE-MARTINERIE C AND JULLIEN P. (1984). Normal embryo fibroblasts release transforming growth factors in a latent form. *J. Cell Physiol.*, **121,** 184–188.

MOORADIAN LD, MCCARTHY BJ, KOMANDURI VK AND FURCHT TL. (1992). Effects of transforming growth factor-$\beta$1 on human pulmonary adenocarcinoma cell adhesions, motility, and invasion *in vitro*. *J. Natl Cancer Inst.*, **84,** 523–527.

MUKAI M, SHINKAI K, KOMATU K AND AKEDO H. (1989). Potentiation of invasive capacity of rat ascites hepatoma cells by transforming growth factor-$\beta$. *Jpn. J. Cancer Res.*, **80,** 107–110.

NAITO Y, KINO I, HORIUTI K AND FUJIMOTO D. (1984). Promotion of collagen production by human fibroblasts with gastric cancer cells *in vitro*. *Virchows Arch. B Cell Pathol.*, **46,** 145–154.

SEKIGUCHI M, SAKAKIBARA K AND FUJII G. (1978). Establishment of cultured cell lines a human gastric carcinoma. *Jpn. J. Exp. Med.*, **48,** 61–69.

SHIBAMOTO S, HAYAKAWA M AND HORI T. (1992). Hepatocyte growth factor transforming growth factor-$\beta$ stimulate both cell growth and mitogen of human gastric adenocarcinoma cells. *Cell Structure Function*, **17,** 185–190.

SHIRASUMA K, MORIOKA S, WATANI K, HAYASHIDO Y, FURUSAWA H, SUGIYAMA M, OKURA M AND MATSUYA T. (1988). Growth inhibition and differentiation of human salivary adenocarcinoma cells by medium conditioned with normal human fibroblasts. *Cancer Res.*, **48,** 2819–2824.

TAHARA E. (1990). Growth factors and oncogenes in human gastrointestinal carcinomas. *J. Cancer Res. Clin. Oncol.*, **116,** 121–131.

TANIMOTO H, YOSHIDA K, YOKOZAKI H, YASUI W, NAKAYAMA H, ITO H, OHAMA K AND TAHARA E. (1991). Expression of basic fibroblast growth factor in human gastric carcinomas. *Virchows Arch. B Cell Pathol.*, **61,** 263–267.

YAMAMOTO R, IISHII H, TATSUTA M, NAKAMURA H, TERADA N, KOMATU K AND MATSUSAKA T. (1990). Enhancement of mucus accumulation in a human gastric scirrhous carcinoma cell line (KATO-III) by fibroblast–tumor cell interaction. *Virchows Arch. B Cell Pathol.*, **59,** 26–31.

YASHIRO M, CHUNG YS AND SOWA M. (1994). Role of orthotopic fibroblasts in the development of scirrhous gastric carcinoma. *Jpn. J. Cancer Res.*, **84,** 883–886.

YASHIRO M, CHUNG YS, NISHIMURA S, INOUE T AND SOWA M. (1995). Establishment of two new scirrhous gastric cancer cell lines: analysis of factors associated with disseminated metastasis. *Br. J. Cancer*, **72,** 1200–1210.

YASUI W, SUMIYOSHI K, HATA J, KAMEDA T, OCHIAI A, ITO H AND TAHARA E. (1988). Expression of epidermal growth factor receptor in human gastric and colonic carcinomas. *Cancer Res.*, **48,** 137–141.

YOSHIDA K, YOKOZAKI H, NIMOTO M, ITO H, ITO M AND TAHARA E. (1989). Expression of TGF-$\beta$1 and procollagen type I and type III in human gastric carcinomas. *Int. J. Cancer*, **44,** 394–398.

YOSHIDA K, KYO E, TUJINO T, SANO T, NIMOTO M AND TAHARA E. (1990). Expression of epidermal growth factor, transforming growth factor-$\alpha$ and their receptor genes in human gastric carcinomas; implication for autocrine growth. *Jpn J. Cancer Res.*, **81,** 43–51.


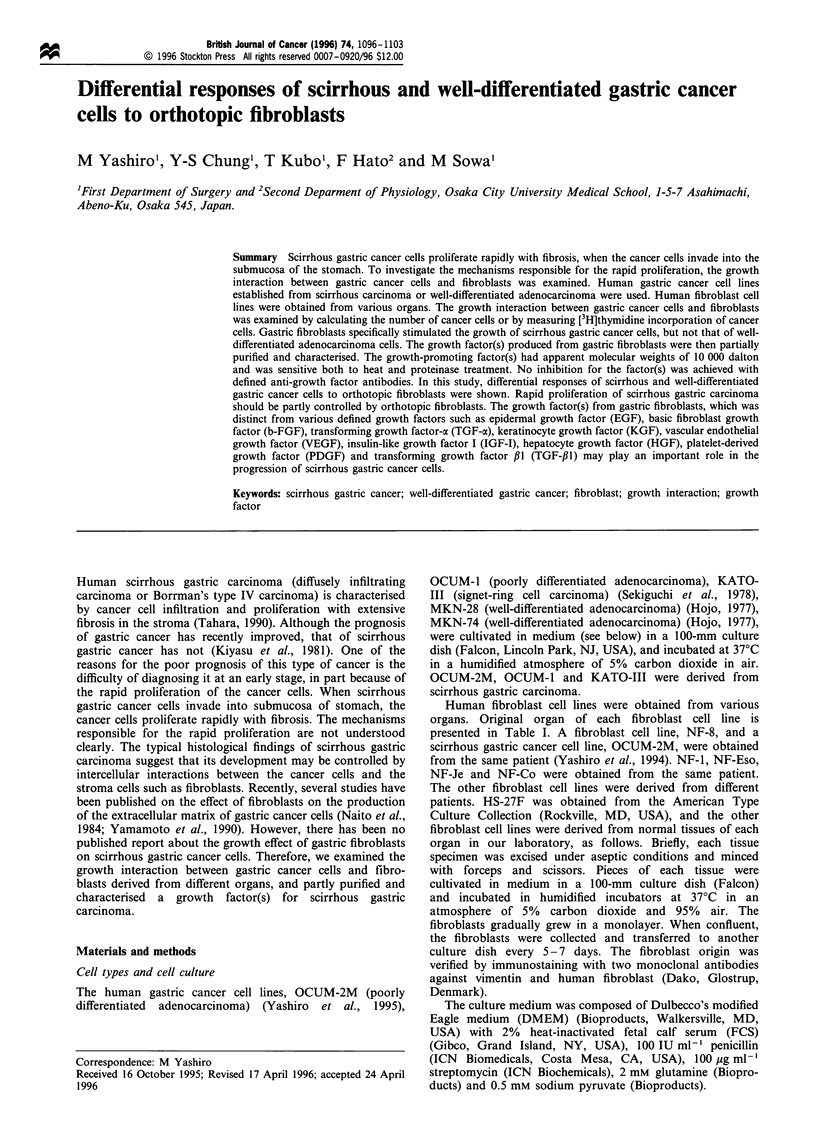

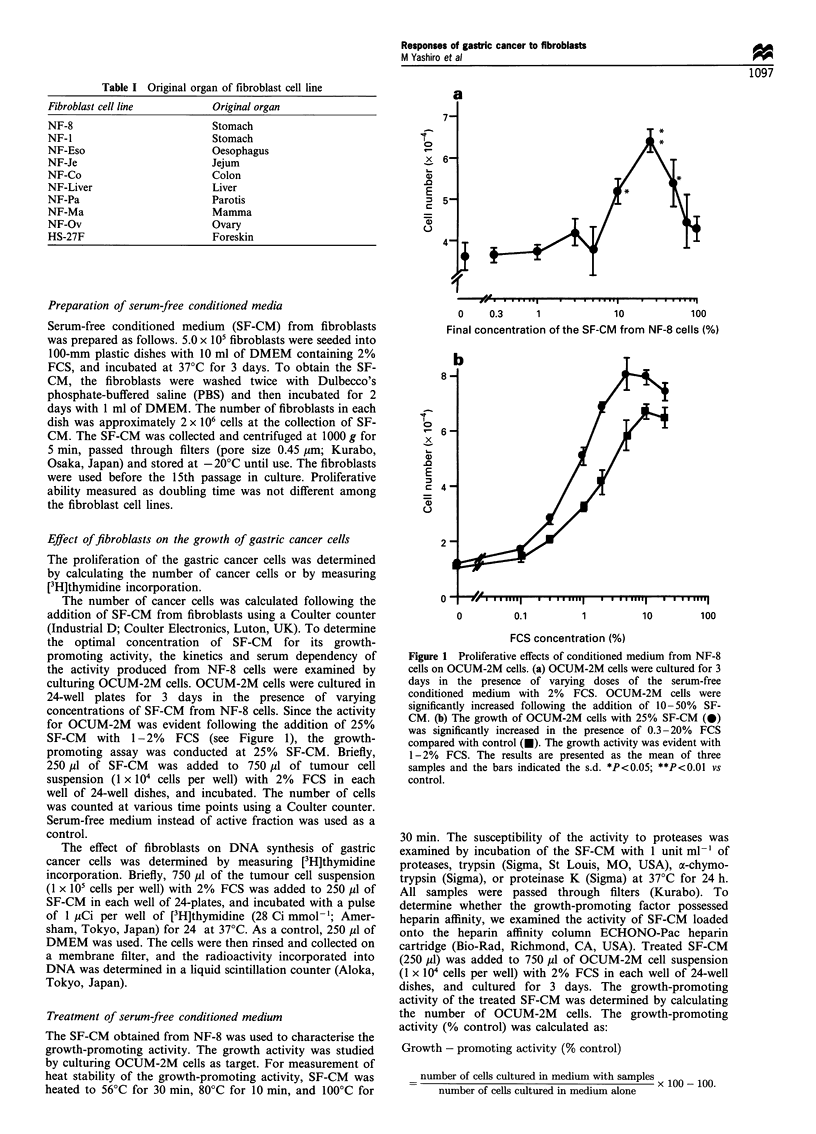

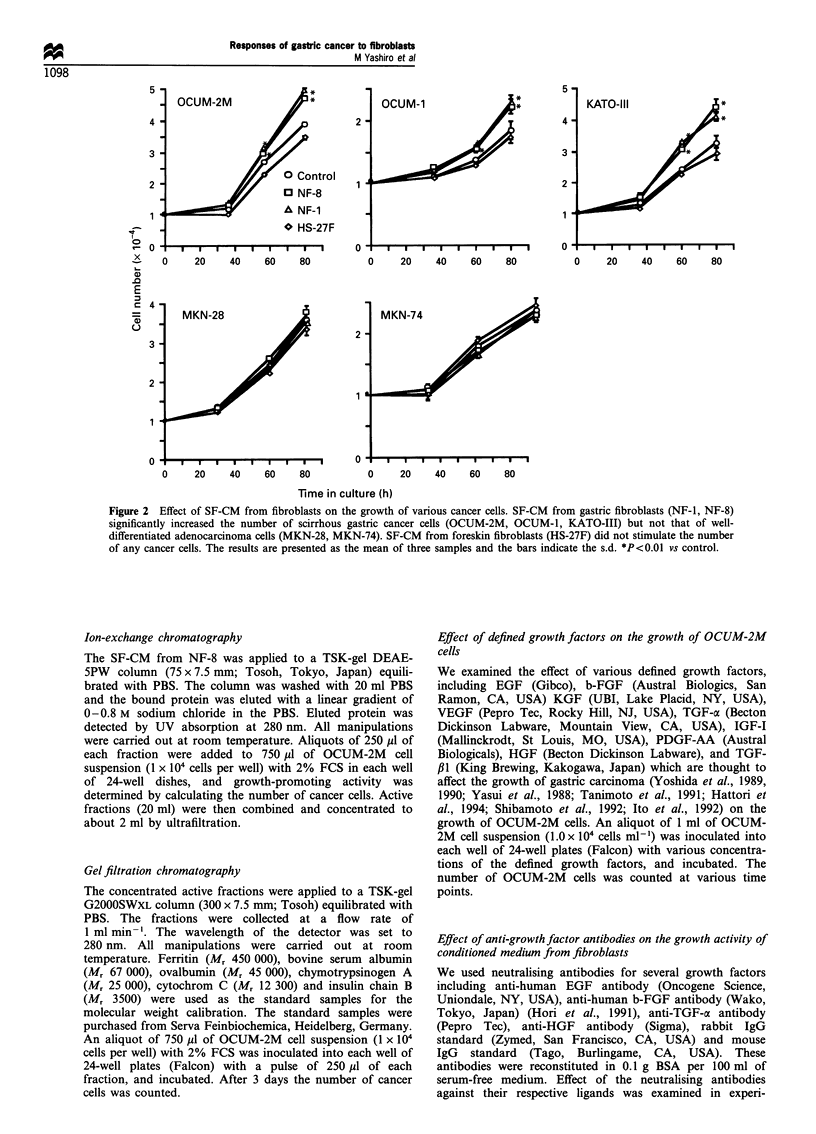

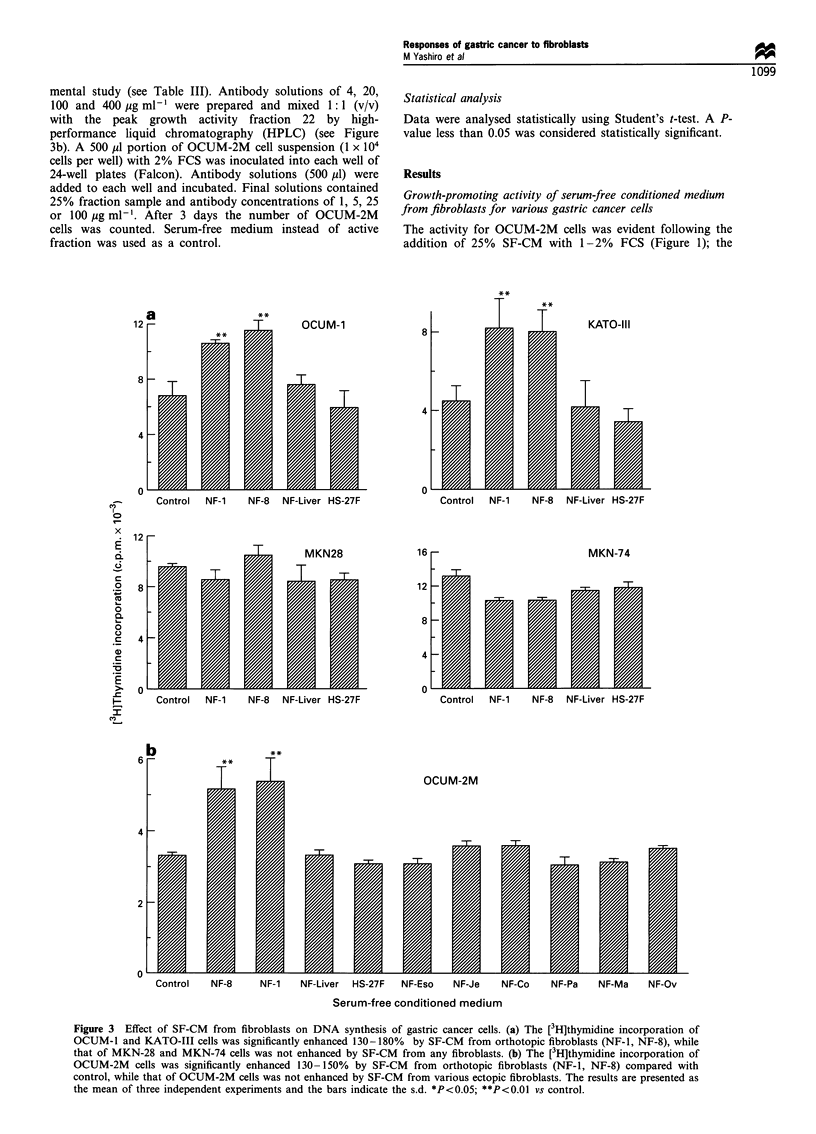

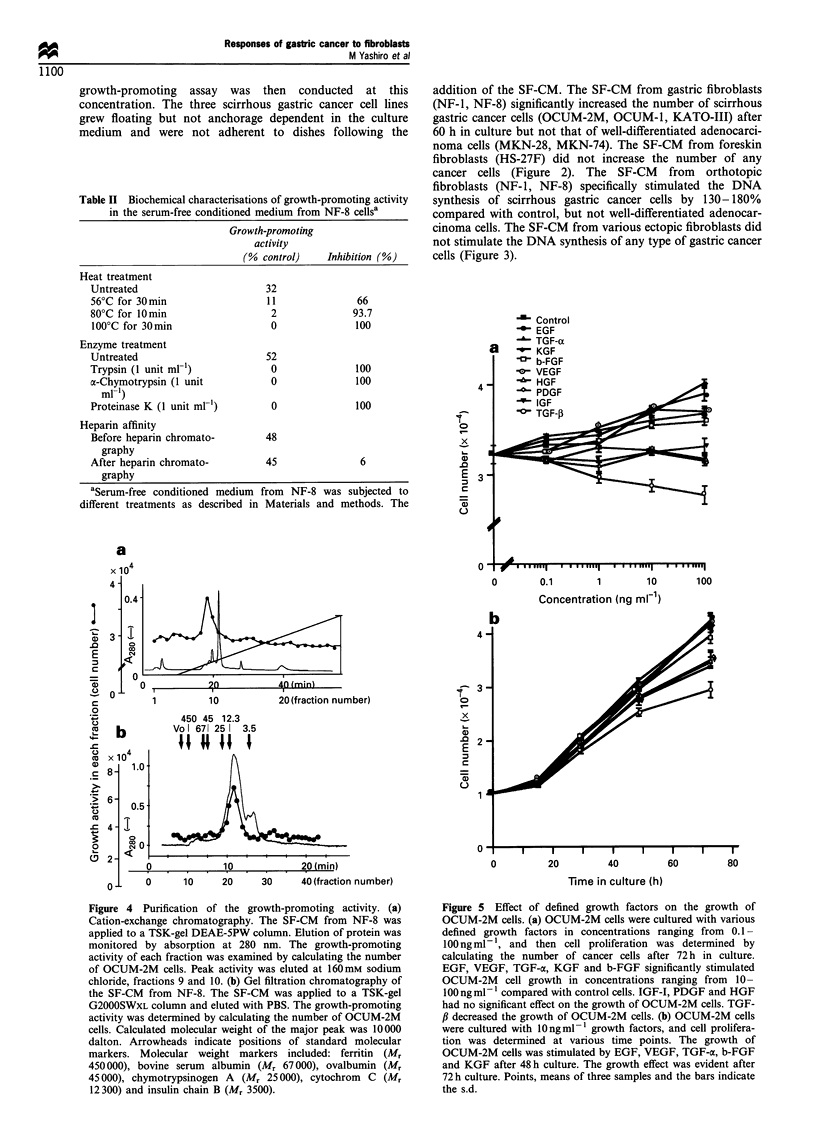

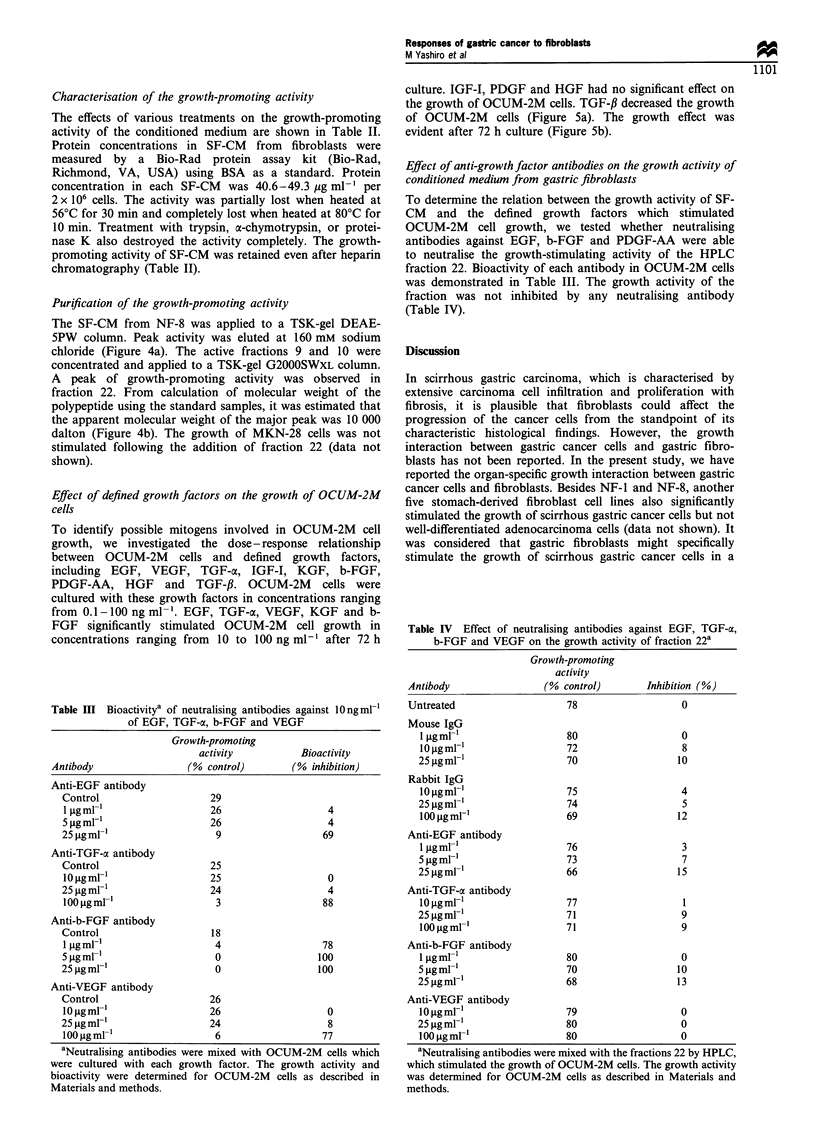

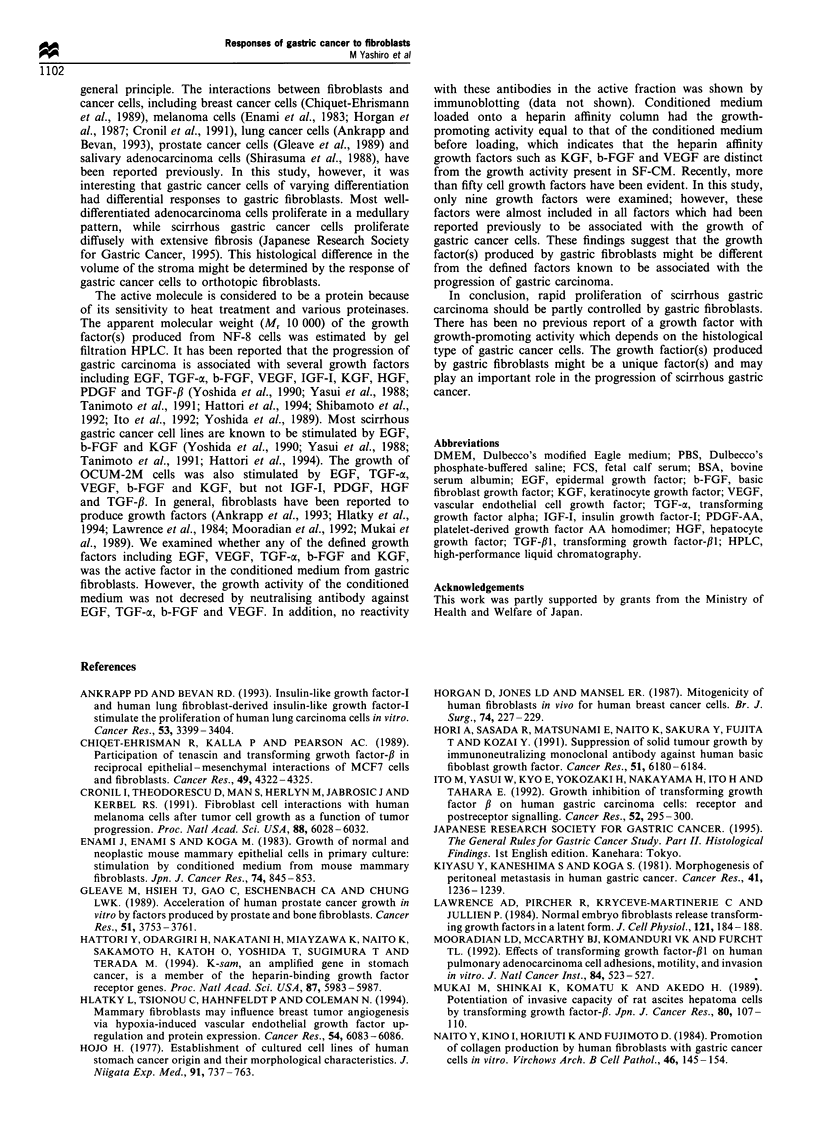

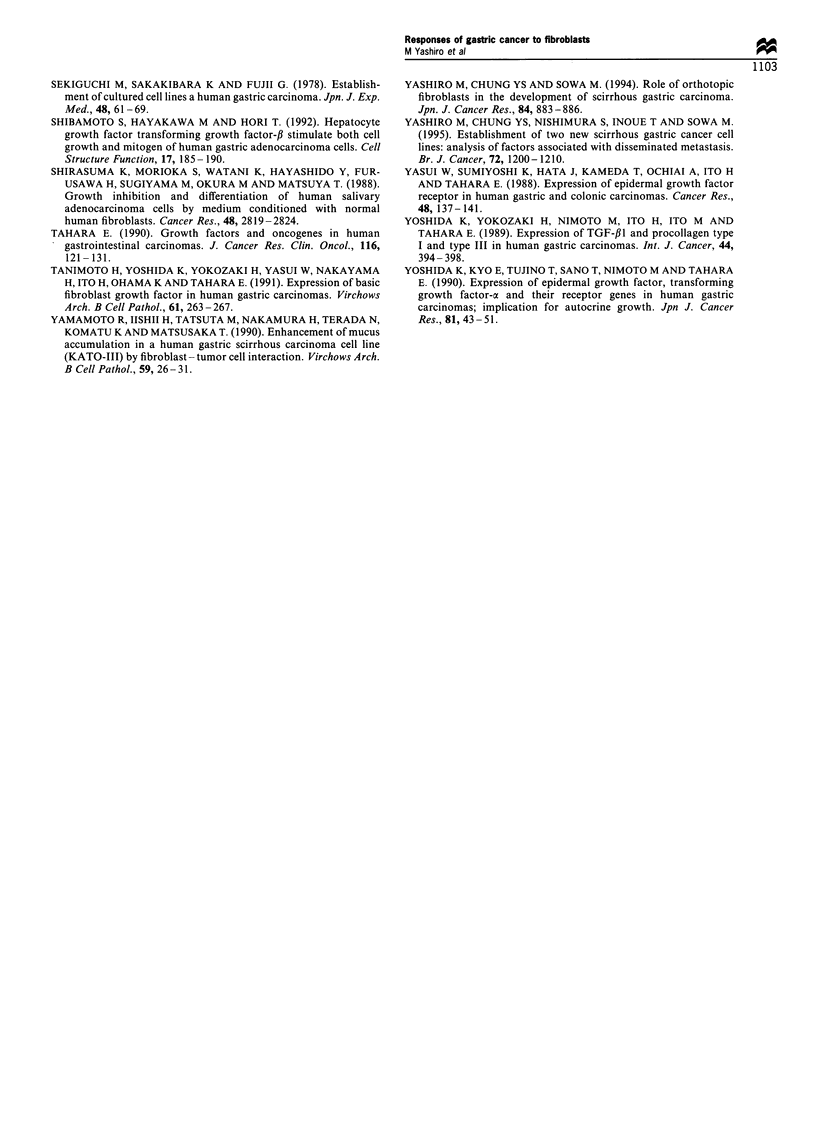


## References

[OCR_01066] Ankrapp D. P., Bevan D. R. (1993). Insulin-like growth factor-I and human lung fibroblast-derived insulin-like growth factor-I stimulate the proliferation of human lung carcinoma cells in vitro.. Cancer Res.

[OCR_01072] Chiquet-Ehrismann R., Kalla P., Pearson C. A. (1989). Participation of tenascin and transforming growth factor-beta in reciprocal epithelial-mesenchymal interactions of MCF7 cells and fibroblasts.. Cancer Res.

[OCR_01078] Cornil I., Theodorescu D., Man S., Herlyn M., Jambrosic J., Kerbel R. S. (1991). Fibroblast cell interactions with human melanoma cells affect tumor cell growth as a function of tumor progression.. Proc Natl Acad Sci U S A.

[OCR_01084] Enami J., Enami S., Koga M. (1983). Growth of normal and neoplastic mouse mammary epithelial cells in primary culture: stimulation by conditioned medium from mouse mammary fibroblasts.. Gan.

[OCR_01090] Gleave M., Hsieh J. T., Gao C. A., von Eschenbach A. C., Chung L. W. (1991). Acceleration of human prostate cancer growth in vivo by factors produced by prostate and bone fibroblasts.. Cancer Res.

[OCR_01096] Hattori Y., Odagiri H., Nakatani H., Miyagawa K., Naito K., Sakamoto H., Katoh O., Yoshida T., Sugimura T., Terada M. (1990). K-sam, an amplified gene in stomach cancer, is a member of the heparin-binding growth factor receptor genes.. Proc Natl Acad Sci U S A.

[OCR_01101] Hlatky L., Tsionou C., Hahnfeldt P., Coleman C. N. (1994). Mammary fibroblasts may influence breast tumor angiogenesis via hypoxia-induced vascular endothelial growth factor up-regulation and protein expression.. Cancer Res.

[OCR_01114] Horgan K., Jones D. L., Mansel R. E. (1987). Mitogenicity of human fibroblasts in vivo for human breast cancer cells.. Br J Surg.

[OCR_01120] Hori A., Sasada R., Matsutani E., Naito K., Sakura Y., Fujita T., Kozai Y. (1991). Suppression of solid tumor growth by immunoneutralizing monoclonal antibody against human basic fibroblast growth factor.. Cancer Res.

[OCR_01123] Ito M., Yasui W., Kyo E., Yokozaki H., Nakayama H., Ito H., Tahara E. (1992). Growth inhibition of transforming growth factor beta on human gastric carcinoma cells: receptor and postreceptor signaling.. Cancer Res.

[OCR_01136] Kiyasu Y., Kaneshima S., Koga S. (1981). Morphogenesis of peritoneal metastasis in human gastric cancer.. Cancer Res.

[OCR_01139] Lawrence D. A., Pircher R., Krycève-Martinerie C., Jullien P. (1984). Normal embryo fibroblasts release transforming growth factors in a latent form.. J Cell Physiol.

[OCR_01145] Mooradian D. L., McCarthy J. B., Komanduri K. V., Furcht L. T. (1992). Effects of transforming growth factor-beta 1 on human pulmonary adenocarcinoma cell adhesion, motility, and invasion in vitro.. J Natl Cancer Inst.

[OCR_01149] Mukai M., Shinkai K., Komatsu K., Akedo H. (1989). Potentiation of invasive capacity of rat ascites hepatoma cells by transforming growth factor-beta.. Jpn J Cancer Res.

[OCR_01157] Naito Y., Kino I., Horiuchi K., Fujimoto D. (1984). Promotion of collagen production by human fibroblasts with gastric cancer cells in vitro.. Virchows Arch B Cell Pathol Incl Mol Pathol.

[OCR_01172] Shibamoto S., Hayakawa M., Hori T., Oku N., Miyazawa K., Kitamura N., Ito F. (1992). Hepatocyte growth factor and transforming growth factor-beta stimulate both cell growth and migration of human gastric adenocarcinoma cells.. Cell Struct Funct.

[OCR_01178] Shirasuna K., Morioka S., Watatani K., Hayashido Y., Furusawa H., Sugiyama M., Okura M., Matsuya T. (1988). Growth inhibition and differentiation of human salivary adenocarcinoma cells by medium conditioned with normal human fibroblasts.. Cancer Res.

[OCR_01185] Tahara E. (1990). Growth factors and oncogenes in human gastrointestinal carcinomas.. J Cancer Res Clin Oncol.

[OCR_01190] Tanimoto H., Yoshida K., Yokozaki H., Yasui W., Nakayama H., Ito H., Ohama K., Tahara E. (1991). Expression of basic fibroblast growth factor in human gastric carcinomas.. Virchows Arch B Cell Pathol Incl Mol Pathol.

[OCR_01196] Yamamoto R., Iishi H., Tatsuta M., Nakamura H., Terada N., Komatsu K., Matsusaka T. (1990). Enhancement of mucus accumulation in a human gastric scirrhous carcinoma cell line (KATO-III) by fibroblast-tumor cell interaction.. Virchows Arch B Cell Pathol Incl Mol Pathol.

[OCR_01208] Yashiro M., Chung Y. S., Nishimura S., Inoue T., Sowa M. (1995). Establishment of two new scirrhous gastric cancer cell lines: analysis of factors associated with disseminated metastasis.. Br J Cancer.

[OCR_01203] Yashiro M., Chung Y. S., Sowa M. (1994). Role of orthotopic fibroblasts in the development of scirrhous gastric carcinoma.. Jpn J Cancer Res.

[OCR_01215] Yasui W., Sumiyoshi H., Hata J., Kameda T., Ochiai A., Ito H., Tahara E. (1988). Expression of epidermal growth factor receptor in human gastric and colonic carcinomas.. Cancer Res.

[OCR_01224] Yoshida K., Kyo E., Tsujino T., Sano T., Niimoto M., Tahara E. (1990). Expression of epidermal growth factor, transforming growth factor-alpha and their receptor genes in human gastric carcinomas; implication for autocrine growth.. Jpn J Cancer Res.

[OCR_01218] Yoshida K., Yokozaki H., Niimoto M., Ito H., Ito M., Tahara E. (1989). Expression of TGF-beta and procollagen type I and type III in human gastric carcinomas.. Int J Cancer.

